# A survey exploring ophthalmologists’ attitudes and beliefs in performing Immediately Sequential Bilateral Cataract Surgery in the United Kingdom

**DOI:** 10.1186/s12886-020-01475-0

**Published:** 2020-06-02

**Authors:** Eunkyung Lee, Bagishan Balasingam, Emily C. Mills, Mehran Zarei-Ghanavati, Christopher Liu

**Affiliations:** 1 Brighton and Sussex Medical School, Brighton, UK; 2 Basingstoke and North Hampshire hospital, Aldermaston road, Basingstoke RG24 9NA, Basingstoke, UK; 3 Farabi Eye Hospital, Tehran University of Medical Sciences, Tehran, Iran; 4 Sussex Eye Hospital, Brighton and Sussex University Hospitals NHS Trust, Eastern Road, Brighton, BN2 5BF UK; 5 Tongdean Eye Clinic, Hove, UK

**Keywords:** Cataract surgery, Sequential bilateral cataract surgery, ISBCS, DSBCS, Cataract, Survey

## Abstract

**Background:**

The standard approach to treat cataracts is Delayed Sequential Bilateral Cataract Surgery (DSBCS), during which patients have a separate operation date for each eye. An alternative method of delivery is Immediately Sequential Bilateral Cataract Surgery (ISBCS). The aim of this project was to examine the attitudes and beliefs of UK ophthalmologists towards ISBCS, explore their reasons to either practise or not practise ISBCS and identify barriers hindering its implementation in the UK.

**Methods:**

A questionnaire was distributed to consultant members of The Royal College of Ophthalmologists (RCOphth, UK) and collected electronically. An initial screening question in regards to prior experience with ISBCS directed the rest of the survey; participants were asked to rate the importance of several factors with regards to performing ISBCS. Free text options were also available. Descriptive analysis was subsequently performed.

**Results:**

Of the 1357 recipients, 130 (9.6%) ophthalmologists completed the survey. Of those, 13.9% were currently performing ISBCS, 83.1% had never performed, and 3.1% had previously done so but since stopped. The main factors that acted as barriers were lack of: (1) College approval (20.5%); (2) medico-legal approval (20.2%); (3) evidence to support the use of ISBCS (16.0%); and (4) hospital approval (13.3%). Additionally, the perceived risk of complications for patients played an important role when considering ISBCS, with the risk of endophthalmitis being most feared.

**Conclusions:**

This survey demonstrates some of the barriers that prevent ophthalmologist’s performing ISBCS in the UK. There is a need for further exploration in this field to evaluate the effect of addressing any of these concerns on the implementation of ISBCS.

## Background

Within the United Kingdom (UK), patients with visually significant cataracts are treated surgically, generally as a day case procedure. Patients can receive their operation state-funded via the National Health Service (NHS) or seek privately funded treatment. In most patients, cataracts eventually occur bilaterally, and the conventional approach for treating bilateral cataracts is Delayed Sequential Bilateral Cataract Surgery (DSBCS) [1]. In this approach, the operation is performed on each eye on different days with an interval that allows surgeons to monitor the outcome in the first eye before proceeding to the second [1]. Cataract treatment is vital, as it is known that restoration of near-normal vision improves quality of life [2, 3]. Recently, a relatively novel approach termed Immediately Sequential Bilateral Cataract Surgery (ISBCS) has been developed [4]. ISBCS involves two eyes to be operated on in the same session, with each eye considered as a separate operation. This will mean re-scrubbing and changing gloves and gowns with each eye as well as using different sterilisation cycles for instruments and implants with different batch numbers or from different manufacturers [4].

The UK National Institute for Health and Care Excellence (NICE), provide further specific criteria to consider when offering ISBCS, which excludes those at increased risk of infections 1. Despite these recognised criteria to exclude high-risk groups, the uptake of ISBCS in the UK is limited in those who are suitable for the procedure. This is in contrast to countries such as Finland where the practice of ISBCS is prevalent [5]. It is clear that there are significant barriers to uptake, which are not fully explained by policy. Barriers to ISBCS within the UK need to be explored, to ensure patients are being offered the correct and appropriate treatment.

The primary aims of this survey was to estimate the prevalence of RCOphth consultant members practising ISBCS. Secondary aims included exploring reasons behind the ophthalmologists’ choice and assessing the findings of this survey in the context of the UK.

## Methods

The questionnaire was formulated from discussions with a local advisory group and other clinical professionals who participated in the practice of ISBCS within Sussex Eye Hospital. It was then externally reviewed by a methodologist at the RCOphth for further modifications and piloted locally prior to dissemination (Additional file 1).

A screening question was used to target specific questions to 3 different groups of consultants: Group 1, who currently perform ISBCS in their clinical practice; Group 2, who have never performed ISBCS; and Group 3, who previously performed ISBCS but no longer do so. The questions in the survey aimed to probe the attitudes and beliefs influencing decision-making processes in consultants. A mixture of questions in the form of multiple-choice, Likert scales and text box entries were included. The questionnaire was peer-reviewed for its accuracy and transferred to Qualtrics (Qualtrics, Provo, UT, Copyright© 2015) a web based software which was used to generate the online survey. The survey was programmed to display the appropriate questions for the category into which each participant fell, based upon their initial screening result.

An invitation email was sent to all consultant ophthalmologists in UK through the Royal College of Ophthalmologists, outlining the aims of the project and explaining how to participate in the online survey. The first page of the survey acted as a participant information sheet and stated responses would be anonymous. Informed consent was obtained upon completion of the survey. The survey remained active for 1 month, and during this time participants were allowed to save and return to their responses at any time. Quantitative data were analysed using descriptive statistical analysis, which was completed by the online survey platform. Qualitative data was not formally analysed but used to support our findings, as such, these findings are presented in Additional file 1.

## Results

Out of 1357 ophthalmologists who were sent the questionnaire, 130 (9.6%) individuals responded. Of these, 18 (13.9%) performed same day sequential bilateral cataract surgery for bilateral visually significant cataracts. In contrast, 108 (83.1%) did not perform same day sequential bilateral cataract surgery, while four (3.1%) had stopped performing same day sequential bilateral cataract surgery.

Of the 18 currently performing ISBCS, one participant reported they had recently started. Seven (38.9%) reported they had done for 2–5 years, and 10 (55.6%) reported the duration to be between 5 and 10 years. Of the 18, the mean percentage of their practice of ISBCS as a total of all cataract operations performed was 15.8%. These respondents also reported that an average of 60% of their eligible patients would proceed to have same day bilateral cataract surgery although there was a wide range in this metric.

Saving patient time was the most important factor for offering ISBCS, with 88.89% of participants reporting this to be very important or important. Saving clinical time and a better visual outcome were reported as very important or important by 47.1 and 47.1% of participants, respectively (Table 1). Of the pre-requisites for ISBCS deemed of note to the participants, no additional risk of endophthalmitis, exclusion of high-risk eyes, the surgeon’s track record, the operating theatre’s infection record, and re-scrubbing and re-gowning before the second eye were rated as either important or very important by over 80% of participants (Table 2).

**Table 1:** Importance ratings of factors that influence the decision to offer ISBCS. The two most important factors were reduced hospital visits and patient convenience

Option	Not important	A little important	Important	Very important
More cost effective for health system	7 (38.9%)	4 (22.2%)	5 (27.8%)	2 (11.1%)
Better visual outcome for patients	4 (23.5%)	5 (29.4%)	3 (17.7%)	5 (29.4%)
Reduces hospital visits for patients, saving their time	1 (5.6%)	1 (5.6%)	7 (38.9%)	9 (50.0%)
More convenient for patients, faster rehabilitation	0 (0%)	2 (11.1%)	5 (27.8%)	11 (61.1%)
Saves more time in clinics and theatre	6 (35.3%)	3 (17.7%)	2 (11.8%)	6 (35.3%)

**Table 2:** Importance ratings of factors that are pre-requisites for ISBCS. A significant amount of importance was given to prevent infection (reducing the risk of endophthalmitis, good infection record and re-gowning and re-gloving)

Option	Not important	A little important	Important	Very important
The patient and their eyes have no additional risk of developing endophthalamitis	0 (0%)	1 (5.6%)	2 (11.1%)	15 (83.3%)
Exclusion of high risk eyes (extremes of axial length, glaucoma, risk of inflammation including cystoid macular oedema, risk of retinal detachment, dense or white nucleus, etc)	1 (5.6%)	2 (11.1%)	5 (27.8%)	10 (55.6%)
Surgeon with a track record	0 (0%)	0 (0%)	10 (55.6%)	8 (44.4%)
Operating facilities have good infection record	0 (0%)	1 (5.6%)	2 (11.1%)	15 (83.3%)
The surgeon and scrub nurse rescrub, regown and reglove before second eye surgery	2 (11.1%)	0 (0%)	0 (0%)	16 (88.9%)
Second surgeon and second scrub nurse scrub for second eye surgery	13 (72.2%)	3 (16.7%)	0 (0%)	2 (11.1%)
Instruments for each operation having gone through different sterilisation cycles	7 (38.9%)	2 (11.1%)	2 (11.1%)	7 (38.9%)
Medicine, solutions and cannulae having come from different manufacturers or have different batch numbers	5 (27.8%)	2 (11.1%)	3 (16.7%)	8 (44.4%)
Day 1 review by ophthalmologist	13 (72.2%)	3 (16.7%)	2 (11.1%)	0 (0%)

Of those who have not performed ISBCS, 22 (16.1%) would do the surgery if it were a surgery for senile cataract under general anaesthesia. Sixty-two (45.3%) reported that they would do such surgery if high-risk general anaesthetic were required. Forty-three (31.4%) participants reported that they would not perform ISBCS for any reasons. One participant reported that they perform same day sequential bilateral cataract surgery for a refractive lens exchange, and one participant reported that they would do it for phakic IOL implantation. Furthermore, eight (5.8%) reported that they would perform it for congenital cataract surgery (Table 3). Some participants stated more than one reason to perform ISBCS.

**Table 3:** List of procedures that participants would be willing to perform ISBCS. A significant portion (31.4%) of participants would not perform ISBCS for any of the procedures listed

Option	Count	Percentage (%)
Refractive lens exchange	1	0.7%
Phakic IOL implantation	1	0.7%
Senile cataract surgery under general anaesthesia (GA)	22	16.1%
Senile cataract surgery under high-risk general anaesthesia (GA)	62	45.3%
Congenital cataract surgery	8	5.8%
I would not do same day sequential bilateral surgery for any of these procedures	43	31.4%
Total	137	100.0%

Of the reasons given for not performing ISBCS, 92.6% of participants rated a risk of endophthalmitis as either very important or important factors when considering offering ISBCS; 54.2, 54.2 and 63.8% of participants rated as either important or very important for the risk of cystoid macular oedema, wrong IOL power and medico-legal issues if surgery is performed incorrectly, respectively (Table 4). When assessing the comments in the free-text box, a number of responses talked about risks including infections other than those mentioned in the question, bleeding, TASS syndrome and PC rupture. Other comments referred to a possible lack of justification for ISBCS, lack of enthusiasm from patients or compliance with post-operative drops. However, many comments related to options already available in the question response list, such as IOL power inaccuracy and endophthalmitis.

**Table 4:** Importance ratings of factors that influence the decision not to perform ISBCS. Risk of endophthalmitis carries a much greater importance to participants compared to other factors. Other reasons not listed can be found in the appendix (Additional file 1)

Option	Not important	A little important	Important	Very important
No evidence of effectiveness	39 (39.4%)	27 (27.3%)	19 (19.2%)	14 (14.1%)
Risk of endophthalmitis	2 (1.9%)	6 (5.6%)	21 (19.4%)	79 (73.2%)
Risk of cystoid macular oedema	22 (20.6%)	27 (25.2%)	33 (30.8%)	25 (23.4%)
Risk of retinal detachment	41 (38.3%)	38 (35.5%)	16 (15.0%)	12 (11.2%)
Risk of wrong IOL power calculation	23 (21.5%)	26 (24.3%)	39 (36.5%)	19 (17.8%)
Risk of other complications- please specify in the box	35 (49.3%)	7 (9.9%)	15 (21.1%)	14 (19.7%)
More familiarity with single eye surgery	68 (66.0%)	16 (15.5%)	10 (9.7%)	9 (8.7%)
Medico-legal issues should same day bilateral cataract surgery goes wrong	13 (12.3%)	25 (23.8%)	31 (29.5%)	36 (34.3%)
I have not been trained to do same day bilateral cataract surgery	91 (86.7%)	7 (6.7%)	4 (3.8%)	3 (2.9%)
Insufficient facilities or support staff	82 (78.9%)	7 (6.7%)	13 (12.5%)	2 (1.9%)
Other reasons- please specify in the box (see Additional file 1)	46 (71.9%)	2 (3.1%)	5 (7.8%)	11 (17.2%)

Of those who have never performed ISBCS, the most important factors that would make them consider bilateral same day sequential cataract surgery were specialist society/College approval (20.5%) and medico-legal/indemnity insurance approval (20.2%). Also, improved evidence of effectiveness and safety were deemed to be important (16.0%). Less important factors were hospital approval (13.3%) and the availability of pre-packed right and left instrument packs to reduce set up time by theatre nurses (4.9%). Factors such as the availability of trained nursing staff (2.3%), improved availability of intracameral cefuroxime (0.8%), and the availability of training for surgeons (0.4%) were deemed to be of little importance (Table 5). When analysing the free text responses, a number of responses made reference to options already present in the questionnaire, as well as issues with peer-acceptance of the procedure, theatre logistics, surgical planning for the second eye based on first surgery outcome, Clinical Commissioning Group (CCG) approval, and patient factors such as general anaesthetic risk, amongst others.

**Table 5:** Importance ratings of factors that influence the decision to consider performing ISBCS. Other reasons not listed can be found in the appendix (Additional file 1)

Option	Count
I would never consider same day sequential bilateral cataract surgery	31 (11.8%)
Improved availability of intracameral cefuroxime	2 (0.8%)
Availability of pre-packed right, and left, instrument packs to reduce set up time by theatre nurses	13 (4.9%)
Trained nursing staff available	6 (2.3%)
Availability of training for surgeon	1 (0.4%)
Improved evidence of effectiveness and safety	42 (16.0%)
Hospital approval	35 (13.3%)
Medico-legal/indemnity insurance approval	53 (20.2%)
Specialist society/College approval	54 (20.5%)
Others- please specify in the box (see Additional file 1)	26 (9.9%)
Total	263

Of the four participants who had stopped performing same day sequential bilateral cataract surgery, two stated financial reasons (e.g. only one procedure is paid for by commissioners, despite two eyes being operated on together). Other reasons included participants no longer believing in the benefits of same day sequential bilateral cataract surgery and restrictions in hospital policy (Table 6).

**Table 6:** Participants’ reasons for stopping ISBCS. Four participants stopped performing ISBCS, with one participant providing two reasons

Option	Count	Percentage (%)
Commissioners only pay for one procedure when the two eyes are done together	2	40.0%
My hospital does not allow routine practice of immediately sequential bilateral cataract surgery	1	20.0%
Peer pressure to stop	0	0.0%
I no longer believe in the benefits of immediately sequential bilateral cataract surgery	2	40.0%
Other reasons- please state in the box	0	0.0%
Total	5	100.0%

## Discussion

In the UK, ISBCS is not widely adopted, which might explain the relatively low respondent rate for our survey. However, it is important that the themes addressed as a result of this questionnaire are explored. From our data, it is also evident that a large proportion of our respondents do not perform ISBCS. Moreover, it was confirmed that some respondents who previously performed ISBCS had stopped, mainly due to financial issues or lack of evidence supporting its use. Less emphasis was put on hospital policy or a lack of College approval. The fact that no respondents reported peer pressure as a barrier might reflect positive attitudes amongst ophthalmologists towards ISBCS.

In regards to specific cases in which ophthalmologists would consider the use of ISBCS, the majority of our participants still reported they would not perform ISBCS under any circumstances, demonstrating a strong resistance towards incorporating it into their clinical practice. However, some stated that they would consider its use. In particular, the use of ISBCS was favoured in the case of patients who required a general anaesthetic as it only requires one episode of anaesthesia. Additionally, it was found that the potential risk of endophthalmitis was the most important factor for participants not to perform ISBCS. This finding was in line with existing literature on reasons for not performing ISBCS [3, 6]. Other complications of ISBCS, such as risk of cystoid macular oedema and risk of an incorrect IOL power were also considered to be important in their decision to practise. Alongside the risk of endophthalmitis, medico-legal issues were feared. Interestingly, a lack of training to perform ISBCS or greater familiarity with single eye surgery was not deemed to be important; the risk of retinal detachment was also not considered to be important. This may suggest that ophthalmologists place a greater emphasis on potential penalties rather than lack of experience.

For those who previously performed ISBCS, a lack of supporting evidence was a significant factor in their decision to stop performing ISBCS. However, it did not impact on the decision to potentially start performing ISBCS for those who had not previously done so.

Several factors that may influence an ophthalmologist’s decision to offer ISBCS have been identified. Patient convenience seems to play a crucial role when considering ISBCS. Many participants reported that they would offer ISBCS as it reduces hospital visits and saves time for travel, as well as providing more convenience and quicker rehabilitation for patients. Some deemed improved visual outcomes for patients to be important. This is importance to note, as it appears that the fears of ophthalmologists may be overshadowing potential benefits for patients.

Looking at our data, it is clear that many ophthalmologists may not perform ISBCS or are reluctant to perform ISBCS in their clinical practice. Despite comparable outcomes to DSBCS demonstrated by high-quality evidence [5, 7], ISBCS is still not adopted as a standard of care to this date.

Previous literature has found that the absence of postoperative refractive outcome from the first eye to guide the lens selection for the second eye, and the risk of bilateral vision loss were among the top concerns [8]. In contrast, our study shows that medicolegal issues and the risk of complications are the most highly rated reasons for not performing ISBCS (Table 4). To address these issues, policies need to be examined and adjusted if necessary, to increase the safe practice of ISBCS [6]. In terms of financial barriers, it is unlikely that ophthalmologists would consider ISBCS with less financial incentive, considering the increased responsibility and tasks to all involved when compared to traditional DSBCS [3]. However, a global solution cannot easily be resolved by changing financial policy. In some settings, the routine practice of ISBCS may not be feasible due to limited resources, especially in developing countries. For example, the use of intracameral antibiotic prophylaxis to prevent endophthalmitis after ISBCS may be limited alongside with a lack of hygiene and operating room protocol among other factors, in which case the risks may outweigh the benefits [9]. Introducing ISBCS to these areas may do more harm than good. Additionally, the risk of complications, especially endophthalmitis, along with other complications was the single most feared factor for our participants, which has been previously noted [6, 10].

Indeed, the majority of complications that are associated with ISBCS are manageable and should not cause unnecessary concern among ophthalmologists. There are published international guidelines^2^ from the International Society of Bilateral Cataract Surgeons, which aim to a minimise the incidence of complications. To our knowledge, the literature to date provides only a small number of case reports of bilateral endophthalmitis following ISBCS, in which none strictly followed the aforementioned guidelines [11–13]. Strict adherence to the protocol should reduce the risk of complications [14]. Some surgeons advocate their own protocols such as not continuing with the second eye the same day if there is an intraoperative complication with the first eye, which may be incorporated. Within the NHS, clear procedural guidelines should be adopted in addition to hospital approval, to change the current culture of resistance towards ISBCS. Despite the theoretical risk of bilateral endophthalmitis being so low, it is an extremely negative outcome for an elective procedure, that may have been avoided by performing delayed surgery. ISBCS may have promising economic benefits [3] but comes with a heightened risk of blindness which patients should be aware of. Some would argue that you cannot justify cost savings that may result in blindness for an individual, albeit exceedingly rare.

ISBCS could potentially provide a better standard of care than DSBCS in cataract operations. During delayed sequential cataract surgery, the second eye is compromised in visual acuity and colour vision whilst waiting for the next operation [15, 16]. Therefore, it may be prudent to operate bilaterally in the first instance in all patients with bilateral cataract [15, 16]. Faster rehabilitation, improved quality of life and less travel time would provide additional benefit to patients [17]. Other advantages for society includes less time off work, reduced hospital resource consumption and efficient use of clinic/operation room time [3]. Patients who are at a high risk of death due to undergoing a second general anaesthetic may also benefit from ISBCS [17]. More research and training is also needed to improve the level of evidence to support the use of ISBCS and to allow ophthalmologists to make a better clinical decision for patients after close examination of its advantages and disadvantages [17].

## Conclusion

Our survey has allowed ophthalmologists to express their views and concerns related to the practice of ISBCS in the UK. Our study also highlights some of the negative factors that need to be overcome for ISBCS to become adopted more widely. Importantly, it provides a basis for which the moral and ethical debates for ISBCS can be discussed. We did not formally statistically analyse our results as it was not within the remit of this survey-based study to explore beliefs and attitudes. Demographics of the participants were not collected and could be incorporated in future surveys to determine whether the age, gender, and professional progression of the participants affect attitudes towards practising ISBCS. It is also worth readdressing that the main limitation of the relatively low response rate of members, will not reflect the views of those who did not reply, so cannot be taken as true representation of all members. However, our study provides a foundation for which we can explore the cultures and practices in other health systems regarding ISBCS. The basis of this survey has already been used to inform a subsequent project to explore the European view on ISBCS [18] and we hope work within this area will broaden into the USA and Asia.

In conclusion, ISBCS has remained a controversial subject and there has been resistance towards its implementation in the clinical practice. From our study, it was evident that a large proportion of ophthalmologists would still not consider practising ISBCS, except in the cases in which patients are at a high risk of complications following a second general anaesthetic. Improved awareness of the practice of ISBCS and college and hospital approval is needed to change the resistant culture of unsubstantiated beliefs towards ISBCS in the world of ophthalmology, especially as it can provide additional benefits to both patients and practitioners compared to DSBCS.

## Supplementary information

**Additional file 1.**

## Data Availability

Available on request (survey responses).

## Abbreviations

UK: United Kingdom
DSBCS: Delayed Sequential Bilateral Cataract Surgery
ISBCS: Immediately Sequential Bilateral Cataract Surgery
RCOphth: Royal College of Ophthalmologists
NICE: National Institute for Health and Care Excellence
IOL: Intraocular Lens
TASS: Toxic Anterior Segment Syndrome
PC: Posterior Capsule
CCG: Clinical Commissioning Group

## Footnotes

1 National Institute of Health and Care Excellence (NICE). Cataracts in adults: management. NICE guideline [NG77]; Oct 2017. Available online: https://www.nice.org.uk/guidance/NG77. Accessed: 2019 Mar 4.

2 International Society of Bilateral Cataract Surgeon (iSBCS). iSBCS General Principles for Excellence in ISBCS 2009. Spain: iSBCS. Available from: http://www.isbcs.org/wp-content/uploads/2011/03/2010-07-20-FINAL-ISBCSSBCS-suggestions-from-ESCRS-Barcelona.pdf. Published: 2009, Spain. Accessed: 2020 Mar 9.
