# Supplementary material for: A survey exploring ophthalmologists’ attitudes and beliefs in performing Immediately Sequential Bilateral Cataract Surgery in the United Kingdom
Source: BMC Ophthalmol. 2020 Jun 2;20:210. doi: 10.1186/s12886-020-01475-0 (PMC7265252; doi:10.1186/s12886-020-01475-0)
Supplement: Supplementary file 1 — Additional file 1. [file 12886_2020_1475_MOESM1_ESM.docx]

| **APPENDIX I. Questionnaires following screening question, development stages shown** | | |
| --- | --- | --- |
| **[Section 1a – I currently practise ISBCS]** | | |
| **Original questionnaire for those answer “YES “to the screening question** | **Questionnaire following clinical expert review** | **Questionnaire following RCOphth** |
| 1) For how long has this been your practice?  (text box entry) | 1) For how long has this been your practice?  (text box entry) | 1) For how long has this been your practice?  🞏Recently started  🞏1-2 years  🞏2-5 years  🞏5 years or longer |
| 2) Roughly what percentage of your cataract operations are bilateral?  🞏0-20%  🞏20-40%  🞏40-80%  🞏80-100% | 2) Roughly, what percentage of your cataract operations are same-day bilateral?  🞏1-20%  🞏21-40%  🞏41-60%  🞏61-80%  🞏81-100% | 2) Please estimate the percentage of your cataract operations that are same-day bilateral?  🞏1-20%  🞏21-40%  🞏41-60%  🞏61-80%  🞏81-100% |
| 3) What are your reasons for considering bilateral cataract surgery? Please rank all of the following which you consider to be important out of 1-5.  🞏More cost effective for health system  🞏Better visual outcome for patients  🞏Reduced hospital visits for patients, saving their time  🞏More convenient for patients, faster rehabilitation  🞏Saves more time in clinics and theatre | 3) What are your reasons for offering same-day bilateral cataract surgery? Please rank all of the following which you consider to be important out of 1-5.  🞏More cost effective for health system  🞏Better visual outcome for patients  🞏Reduced hospital visits for patients, saving their time  🞏More convenient for patients, faster rehabilitation  🞏Saves more time in clinics and theatre | \|  \| Not important \| Quite important \| Important \| Very important \| \| --- \| --- \| --- \| --- \| --- \| \| More cost effective for health system \| 🞏 \| 🞏 \| 🞏 \| 🞏 \| \| Better visual outcome for patients \| 🞏 \| 🞏 \| 🞏 \| 🞏 \| \| Reduced hospital visits for patients, saving their time \| 🞏 \| 🞏 \| 🞏 \| 🞏 \| \| More convenient for patients, faster rehabilitation \| 🞏 \| 🞏 \| 🞏 \| 🞏 \| \| Saves more time in clinics and theatre \| 🞏 \| 🞏 \| 🞏 \| 🞏 \|   3) What are your reasons for offering same-day bilateral cataract surgery? Please rank the importance of the following: |
| Have your patients encountered any complications following bilateral cataract surgery?  🞏Endophthalamitis  o Bilateral  o Unilateral  🞏Bilateral cystoid macula oedema  🞏Bilateral retinal detachments  🞏Bilateral wrong IOL calculation  🞏Other | Have any of your patients encountered the following complications following same-day bilateral cataract surgery?  🞏Unilateral endophthalamitis  🞏Bilateral endophthalamitiis  🞏Bilateral cystoid macular oedema  🞏Bilateral retinal detachment  🞏Bilateral significant refractive surprise  🞏Other significant complications, please state …………………………………………………………………………… | Question removed |
| 4) What are your pre-requisites for performing bilateral cataract surgery?  🞏Patient has no risk of infection  🞏No high risk eyes  🞏Surgeon has a track record  🞏The surgeon and assistant rescrub before second eye operation  🞏Another surgeon and assistant scrub as the first eye operation comes to an end  🞏Instruments obtained from a “phaco pack” held back from the previous week's  batch  🞏same day follow up by ophthalmologist before discharge  🞏others, please state | 4) What do you consider to be pre-requisites for same-day bilateral cataract surgery?  🞏The patient and their eyes have no additional risk of developing endophthalamitis  🞏Exclusion of high risk eyes (extremes of axial length, glaucoma, risk of inflammation including CMO, risk of retinal detachment, dense or white nucleus, etc.)  🞏Surgeon has a track record  🞏Operating facilities have good infection record  🞏The surgeon and scrub nurse rescrub, regown and reglove before second eye surgery  🞏Second surgeon and second scrub nurse scrub for second eye surgery  🞏Instruments for each operation having gone through different sterilisation cycles  🞏Medicine, solutions and cannulae having come from different manufacturers or have different batch numbers  🞏Day 1 review by ophthalmologist  🞏Others, please state …………………………….. | 4) How important do you think the following pre-requisites for same-day bilateral cataract surgery?   \| **Pre-requisites** \| **Not important** \| **Quite Important** \| **Important** \| **Very important** \| \| --- \| --- \| --- \| --- \| --- \| \| The patient and their eyes have no additional risk of developing endophthalamitis \| 🞏 \| 🞏 \| 🞏 \| 🞏 \| \| Exclusion of high risk eyes (extremes of axial length, glaucoma, risk of inflammation including CMO, risk of retinal detachment, dense or white nucleus, etc.) \| 🞏 \| 🞏 \| 🞏 \| 🞏 \| \| Surgeon has a track record \| 🞏 \| 🞏 \| 🞏 \| 🞏 \| \| Operating facilities have good infection record \| 🞏 \| 🞏 \| 🞏 \| 🞏 \| \| The surgeon and scrub nurse rescrub, regown and reglove before second eye surgery \| 🞏 \| 🞏 \| 🞏 \| 🞏 \| \| Second surgeon and second scrub nurse scrub for second eye surgery \| 🞏 \| 🞏 \| 🞏 \| 🞏 \| \| Instruments for each operation having gone through different sterilisation cycles \| 🞏 \| 🞏 \| 🞏 \| 🞏 \| \| Medicine, solutions and cannulae having come from different manufacturers or have different batch numbers \| 🞏 \| 🞏 \| 🞏 \| 🞏 \| \| Day 1 review by ophthalmologist \| 🞏 \| 🞏 \| 🞏 \| 🞏 \| |
| 5) After an informed discussion, how many patients accept to have bilateral surgeries?  🞏Very few cases  🞏Many cases  🞏Half the cases  🞏Most | 5)For patients you consider suitable for same day bilateral cataract surgery, following informed discussion, what percentage actually go on to have same-day bilateral surgery?  🞏1-25%  🞏26-50%  🞏51-75%  🞏76-100% | 5) For patients you consider suitable for same day bilateral cataract surgery, following informed discussion, what percentage actually go on to have same-day bilateral surgery?  🞏1-25%  🞏26-50%  🞏51-75%  🞏76-100% |

| **[Section 1b- I do not practise ISBCS]** | | |
| --- | --- | --- |
| Original NO questionnaire | Questionnaire following clinical expert review | Questionnaire following RCOphth review |
| 1) Would you perform a bilateral cataract surgery on these patients?  🞏On patients with local anaesthetic?  Yes or no?  🞏On patients with general anaesthetic?  Yes or no? | 1) Would you offer simultaneous (immediately sequential) same-day bilateral surgery for the following procedures?  🞏Refractive lens exchange  🞏Phakic IOL implantation  🞏Senile cataract surgery under general anaesthesia (GA)  🞏Senile cataract surgery under high risk general anaesthesia (GA)  🞏Congenital cataract surgery | 1) Would you do simultaneous (immediately sequential) same-day bilateral surgery for the following procedures? Please tick all that apply.  🞏Refractive lens exchange  🞏Phakic IOL implantation  🞏Senile cataract surgery under general anaesthesia (GA)  🞏Senile cataract surgery under high risk general anaesthesia (GA)  🞏Congenital cataract surgery |
| 2) What are the reasons for not considering bilateral cataract surgery? Please select all that apply.  🞏 No evidence of effectiveness  🞏Surgical risks e.g intraocular infections  🞏Familiarity with single eye surgery  🞏Medico-legal issues if it goes wrong  🞏No training  🞏Other | 2) What are your reasons for not offering bilateral same-day cataract surgery?  🞏No evidence of effectiveness  🞏Risk of complications  🞏Endophthalmitis  🞏Wrong IOL power calculation  🞏Other complication(s) ………………………  🞏Familiarity with single eye surgery  🞏Medico-legal issues should same-day bilateral cataract surgery goes wrong  🞏I have not been trained to do same day bilateral surgery  🞏Other reason(s) ……………………………. | \|  \| Not Important \| Quite important \| Important \| Very Important \| \| --- \| --- \| --- \| --- \| --- \| \| No evidence of effectiveness \| 🞏 \| 🞏 \| 🞏 \| 🞏 \| \| Risk of Endophthalmitis \| 🞏 \| 🞏 \| 🞏 \| 🞏 \| \| Risk of Cystoid macular oedema \| 🞏 \| 🞏 \| 🞏 \| 🞏 \| \| Risk of Retinal detachment \| 🞏 \| 🞏 \| 🞏 \| 🞏 \| \| Risk of Wrong IOL power calculation \| 🞏 \| 🞏 \| 🞏 \| 🞏 \| \| Risk of Other complications (Please specify)……………………… \| 🞏 \| 🞏 \| 🞏 \| 🞏 \| \| Familiarity with single eye surgery \| 🞏 \| 🞏 \| 🞏 \| 🞏 \| \| Medico-legal issues should same-day bilateral cataract surgery goes wrong \| 🞏 \| 🞏 \| 🞏 \| 🞏 \| \| I have not been trained to do same day bilateral surgery \| 🞏 \| 🞏 \| 🞏 \| 🞏 \| \| Insufficient facilities or support staff? \| 🞏 \| 🞏 \| 🞏 \| 🞏 \| \| Other reason(s) ……………………………………….. \| 🞏 \| 🞏 \| 🞏 \| 🞏 \|   2) How important are each of the following reasons for not doing same day sequential bilateral cataract surgery? |
| 3) When would you consider bilateral same day sequential cataract surgery? Please select all that apply.  🞏Availability of the resources  🞏If you had more training  🞏More evidence to show it’s safe and effective  🞏In high risk general anaesthetic patients  🞏Other………………………………… | 3) What would need to happen before you could consider bilateral same-day cataract surgery?  When the following resources are available: 🞏Intracameral cefuroxime  🞏 Pre-packed right and left eye instrument packs  🞏 Trained nursing staff  🞏Training for surgeon  🞏If there was more evidence to show its safe and effective  🞏Hospital approval  🞏Medico-legal / indemnity insurance approval  🞏Specialist society / College approval  🞏Other(s) …………………………….. | 3) Please indicate the factors that would influence your decision to consider bilateral same-day cataract surgery? (please tick all that apply)  🞏 I would not consider bilateral same-day cataract surgery  🞏 Improved availability of Intracameral cefuroxime  🞏 Ability to use specific purpose pre-packed right and left eye instrument packs  🞏 Trained nursing staff available  🞏 Training for surgeon  🞏 Improved evidence of effectiveness and safety  🞏 Hospital approval  🞏 Medico-legal / indemnity insurance approval  🞏 Specialist society / College approval  Others:…………………………………………………………………………… |

| **[Section 1c- Previously Did So]** | | |
| --- | --- | --- |
| Original questionnaire for surgeons selecting “I previously did so”: Contained all of the above for those who don’t perform ISBCS and the following below. | “I previously did so” Questionnaire following clinical expert review | “I previously did so” Questionnaire following RCOphth |
| Please mention the reason(s) for stopping  ………………………………………………….. | Please mention the reason(s) for stopping  ………………………………………………….. | **What is the reason(s) for stopping: please tick all that apply.**  🞏 Commissioners only pay for one procedure when the two eyes are done together  🞏 My hospital does not allow routine practice of bilateral cataract surgery  🞏 Peer pressure to stop  🞏 I no longer believe in the benefits of immediately sequential bilateral cataract surgery  🞏 Other reason-please state: ............................................. |

**Appendix II.**

Appendix 2

**Appendix 2.1.** Other reasons given by participants to not perform ISBCS.

| Risks involved in performing ISBCS | | |
| --- | --- | --- |
| Patient acceptance of risks | Any unforeseen systematic problem | Corneal decompensation |
| Flawed statistical reasoning on risks | Delayed suprachoroidal haemorrhage | Temporary bilateral visual disability |
| Any risk | Suprachoroidal haemorrhage | Blindness |
| Patient unable to function | Wound leak, uveitis | PC Rupture +/- Vitreous Loss +/- Lens Drop |
| Repeated GA risk | TASS, fluid contamination | The effects of bilateral visual loss for the patient should there be endophthalmitis or CMO. Also catastrophic consequences if unforeseen systematic problem passed on to patient in so doing. Surgeons who do this tend to be near pole position of the more arrogant end of the egotistical/narcissistic spectrum |
| Perceived lack of justification | | |
| Why do it- only help is admin and patient satisfaction. If so debilitated to need, maybe one eye is sufficient. | No need or justification. An elderly senile case under GA should achieve benefits from single eye surgery. I would expect there to be an argument for congenital cataract surgery, but I do not perform and have minimal experience of this | Very few others doing it |
| Inappropriate patient selection and unproven audited methodology in unit practicing this delivery | Herd instinct. No one else doing it | I offer to patients what I would prefer to have on my eye |
| Accuracy of IOL power | | |
| Dysphotopsia, refractive surprise with correct IOL power calculation or other dissatisfaction | Accuracy of first eye IOL calculations.  Optimizing refractive outcome for second eye and rehabilitation better with one then the second | Modulating IOL choice 2^nd^ eye with 1^st^ eye refraction |
| Technical/logistical issues | | |
| Complexity of having to use different batches for everything | Having theatre staff being aware of different batch disposable (E.g viscoelastics) | Inequality of access for patients who have not had any eye done and long waiting lists |
| Only really justifiable if the patient is so ill they cannot survive two procedures, and then, a double operation is likely to be too long | In same time it takes to operate on one patient, you could have operated on two. With limited resources it is more sensible to give two patients good vision in the time it takes to give only one patient good vision if it is a bilateral surgery | |
| College/GMC guidelines | Patient Factors | Financial reason |
| NHS and RCOphth policies | Non-compliance with drops | No real incentive particularly privately |
| No College guidelines | Lack of enthusiasm from patients | Reimbursement |

**Appendix 2.2** Other factors that influence the decision to consider ISBCS.

| Patient factors/risk | | |
| --- | --- | --- |
| Patient specific issues | Patient need or it is the best interest of the patient | Appropriate risk factors. Dementia and poor anesthetic risk |
| If there was a strong medical reason, I would consider it maybe | Medically necessary to do so | Security that bilateral vision loss would not take place |
| Anaesthetic risk | | |
| Only high-risk GA patients | Only where multiple GA are contraindicated strongly | Would only consider if it is high risk GA cases |
| Happy to do this for patients requiring GA | I only do bilateral surgery if the patient requires a GA and there would be significant risk to patient from having two GA’s rather than one due to exceptional anesthetic risk factors. Otherwise, I do not consider it to be justified at any time | |
| Preference/Peer pressure | | |
| I do not wish to perform it more widely than I do at present | All of the above would be necessary in the event of doing bilateral surgery, but even if available, I would not contemplate bilateral surgery | If everyone else was doing it, I would feel safe. |
| Lack of evidence | | |
| Refractive audit outcomes to compare bilateral outcomes against unilateral outcomes | Flies in the face of common sense whatever support active data may be dredged up | Evidence of cost-effectiveness |
| Logistical/technical issues | | |
| I would never say never, but I have never had a case where I have felt that it is necessary for patient to have bilateral surgery on the same day. I have however had plenty of patients where their feedback following the first eye surgery for all sorts of reasons has altered the way in which I plan surgery for the second. | Logistics of theatre supplies to be duplicated with different batch numbers of drugs and instrumentation | With such a long waiting list, I would like to rehabilitate as many patients as possible quickly by doing at least one eye |
| Lack of guidelines/approval | | |
| Agreed criteria and consent process indicating patient understanding, recognized diploma or certificate of approval for surgeon and/or unit that they conform to best practice (E.g complete separateness for each eye procedure) | CCG approval | |

**Appendix 2.3** Free comments from participants.

| Comments that are unsupportive of ISBCS |
| --- |
| •I do it rarely.  • Ridiculous idea.  • I have done it before, when young and impressionable, but would not do it again.  • Seldom do this. Only if patient requests, or patient needs a GA because of anxiety or unable to cooperate.  • Would be very very reluctant to do it. Happy with 5-7 days between eyes. Safe and works well.  • It needs to be considered very carefully as it is elective surgery where risks outweigh the benefits.  • Should not be done.  • Difficult to justify and very difficult for the patient if any complications to cope with both eyes recovery.  • There is little or no justification and the legal implications are very significant.  • People are not numbers on a spreadsheet.  • I think it is crazy to perform it. As I understand it, it is virtually never done in the USA.  • Advantageous in patients at risk of anisometropia. I tried to implement this but was told only one procedure at a time could be on the waiting list and consultant colleagues did not share my views in the advantages in reducing falls etc in this group of patients.  • Cataract surgery is an elective procedure and there is no need to increase risk by performing bilateral same day sequential cataract surgery.  • I cannot see the benefit... only a risk, small as it may be, it is still there.  • Why perform two elective procedures on same day if there is even the smallest risk of bilateral blindness? Makes no sense.  • Totally unnecessary high risk for no gain time-consuming unfair on first eye patients.  • No need for this. Risk to patient of catastrophic outcome unnecessary.  • This surgery increases the risk of bilateral blindness due to endophthalmitis to close to 1 in 1000 rather than 1/1000 * 1/1000 = 1/1000000. I would not be prepared to contemplate this risk to my patients though understand that some surgeons might.  • The risks are minimal to negligible but the consequences of contaminated batches of drugs or failure in an element of theatre sterility resulting in bilateral blindness put me off.  • Supporters say that risk of infection for 1 eye in 1 in 1,000. Hence risk for 2 eyes is 1 in 1 million. They apply the same reasoning for other risks, such as retinal detachment, CMO etc. In reality, there are other factors other that 'chance' that determine whether a patient will develop a complication or not. Often, there are inherent pre-existing factors that determine whether a patient will develop a complication or not, e.g. contaminated solutions, instruments, patient factors etc which may lead to cluster' effects. Same applies for CMO. If a patient has CMO in one eye, then the risk is much higher in the other eye. The clinician is then left in the position of dealing with a complication in both eyes at the same time, which can be very devastating for the patient, especially if the vision was quite good before the operation. At least, if we do only one eye first and the patient develops a complication in that eye, the patient and the surgeon can 'pause' and reconsider their options for the 2nd eye.  • The only benefit is reduced cost. With an efficient unit doing the second eye two weeks later is more acceptable to the patient and doesn't require any additional cost.  • I don't think there's much benefit either way. I don't think this is an important question especially when there are truly important questions to be answered. Also not suitable in Toric implants or monovision.  • I think that, if it became more widely accepted, patients would be put under pressure to agree to it as a precondition for the NHS paying for second eye surgery.  • The risks are borne by secondary care/surgeons - the benefits are all for the patient, their family, primary care (eg district nurses) - hence we will never do it as the people who decide carry the risk but get almost none of the benefit. If patients were allowed to choose - they would definitely choose it - much more convenient.  • I feel not relevant, as every person should have access to first eye surgery before considering this approach.  • For me is only available for private patients currently, due to unfavourable Payment by Results.  •My own view is that potential economic or other benefit of this is outweighed by very rare risk of avoidable immediate sudden bilateral blindness.  • Tariff for one eye the same as for bilateral - but costs almost double.  • I frankly think it a bit nuts. I wouldn't want for my cat let alone self or family. I occasionally do it for GA cases to help to avoid a second general anaesthetic typically for those cognitively impaired who a second GA is definitely not in their best interests - but this is less than 1% of my adult cataract practice. |

Appendix 2.4

| Comments that are conditionally or fully supportive of ISBCS |
| --- |
| • Should be offered routinely to all patients.  • Popular with my local patients in the west of Scotland. Many have the inconvenience of long journeys from remote parts of Scotland.  • I only do this for private patients, it works out cheaper for them. I always make sure that I document the discussion on bilateral endophthlamitis & bilateral refractive surprise. In NHS we are only allowed to do bilateral if GA (indeed, I get the impression that we're encouraged to do bilateral if it is a GA)  • Patients and their carers like it.  • It may be appropriate in patients who need GA but are high risk for GA or those with mental capacity issues. Though, I would try and avoid due the potential risk of endophthalmitis.  • Only used in exceptional circumstances ie patient needs GA for surgery and 2 GAs contraindicated.  • I only do same day bilateral surgery under GA for patients with Dementia - to avoid 2 GA's.  • I only perform bilateral sequential cataracts on patients with learning difficulties or those that lack capacity and even with these only in suitable cases where it would be better for both the patient and carers to have both surgery completed at the same time. One GA, less post-operative support, carers, family, district nurse etc.  • Important where a 'best interest' decision has been made on surgery for a patient lacking capacity.  • I only use it when there is a clear need for bilateral surgery and there are risks to the patient with the type of anaesthesia that they require such as a GA.  • I do it in cases: dementia needing GA; any other pathology needing GA; high myopia with risk of anisometropia, patient choice as being busy with life.  • Yes, I only do them for patients who are elderly and have to travel from afar (up to 300 miles!) for each surgery. On balance, it seems very successful and popular for the few that I do and I feel we should be doing more. Very quick, very easy, very efficient.  • Have only done once for patient with learning difficulties high risk GA.  • I only do this in a patient who is severely incapacitated and is under general anaesthetic.  • I do not offer bilateral immediately sequential surgery except in GA cases where risk of a 2nd GA in my opinion outweighs risk of surgical complications. For patients with high refractive errors, I do offer 2nd eye surgery four weeks later and in some circumstances a week later.  • I only do simultaneous bilateral surgery if the patient would not tolerate/ be able to cooperate with local anaesthesia, is not well enough to tolerate a second anaesthetic. All disposables are from different batches/companies for each eye, vancomycin for one eye cefuroxime for the other and different implants for each eye.  • May consider it in congenital cataract in a child.  • Nice thing to do for the patient especially very elderly.  • This is something I might do once in a blue moon but bizarrely I did one yesterday. Basically it is where the patient has to have a GA & they might be ASA 3 or something. Just seems kinder & safer to avoid 2 GAs. I would never do it under local as use subTenons LA.  •Supporting evidence for effectiveness and cost effectiveness needed for this approach to be adopted.  • Very hesitant to do it but happy to explore possibility if strongly supported. Also, bilateral cataract surgery is not what I would consider for supervising a trainee learning or improving cataract surgery.  • Good evidence that it is cost effective, but tariffs would need to change to make it attractive financially. Potential effects on patients most important.  • Not able to offer this for NHS or some insured private patients otherwise would do it on most patients - I believe in most cases it is a superior option.  • Most patients perceive there first eye as a WOW factor, the second eye most of the time they are disappointed with, it just helps a little, thus there is no rush to do both at same time. Reimbursement fees under NHS and private are also less so no incentive for hospitals or surgeons. This is a big issue. It needs NICE approval, college approval and indemnity. And shown to be more cost effective.  • Given waiting times and lack of prioritisation/urgent need of second eye surgery it is a bit of an anathema. |

Appendix 2.5

| Miscellaneous Comments |
| --- |
| • It should be emphasised that not only treat each eye as a separate case (rescrub, etc) but also different batch numbers for kit is essential.  • I perform bilateral surgery using the femtosecond laser, not manual phako.  • Thanks - I have taken on a number of bilateral sequential cases and audited them. All were GA and we looked specifically at our adherence to protocol re separate ops, scrub batches etc. I only took on after discussion and clearing with colleagues so as not to be a maverick. All the reading I did suggested lower risk endophthalmitis etc presumably bilateral surgeons may be more particular knowing the concerns? I joined the special interest group and have spent time personally accrediting myself- and insisting on training the nurses and briefing all in theatre at beginning of a relevant list. I don't think all patients could cope with bilateral surgery, but if at outpatient department a patient expresses interest, is predictably straightforward and in the face of rationing, unnecessary between eye delays affecting anisometropia, spectacle acquisition I believe this is an option I would be prepared to explore. I do not think it suitable for all surgeons or for pooled lists; surgeons need to have continuity of care for consent and agreement of the rules, and adherence to protocol. In the face of current risk of only eye being done on NHS or obfuscatory inter-eye delays not in patient interest the risk benefit ratio may have changed. The economic arguments are about reduced surgeon remuneration and an American issue- not sure about England/ Wales - but in old fashioned Scotland it may offer economic advantage by reducing outpatient department and adherence to waiting times targets. However, safety is the main concern and with advent of disposable gear, small incision, intracameral antibiotics, and strict adherence to known standards of care and best practice, the patient may have a quicker better outcome without the extra six months between eyes!  • It convert independent risk into related risk - only justifiable when offset against another risk.  • Cases would have to be chosen carefully, and consent meticulously documented.  • Would be good to see some evidence / guidance from the Royal College.  • Should comply with iSBCS guidelines.  • In an NHS setting of second eye cataract questionnaires being required, the sequential surgery question is never raised.  • I think it seriously needs to be debated and considered.  • Culture change is beginning and will happen full scale within five to ten years.  • Very important survey, good luck! |
